# Supplementary material for: Hypoplastic and Congenital Absence of Coronary Arteries and Its Correlation with Clinical Implications of Cardiac Circulation: A Systematic Review and Meta-Analysis
Source: J Clin Med. 2024 May 24;13(11):3085. doi: 10.3390/jcm13113085 (PMC11173156; doi:10.3390/jcm13113085)
Supplement: Supplementary file 1 [file jcm-13-03085-s001.zip › jcm-2994309-supplementary.pdf]

Complementary Table S1. Search details.

Database	Search strategy	Results	
		20-03-24	
Medline	("abnormalities"[MeSH Subheading] OR "abnormalities"[All Fields] OR "agenesis"[All Fields] OR "agenesy"[All Fields]) AND ("coronaries"[All Fields] OR "heart"[MeSH Terms] OR "heart"[All Fields] OR "coronary"[All Fields]) AND ("hypoplastic"[All Fields] AND ("coronaries"[All Fields] OR "heart"[MeSH Terms] OR "heart"[All Fields] OR "coronary"[All Fields])) AND ("clin anat"[Journal] OR ("clinical"[All Fields] AND "anatomy"[All Fields]) OR "clinical anatomy"[All Fields])	31	
SCOPUS	((agenesis coronary) AND (hypoplastic coronary)) AND (clinical anatomy)	20	
Lilacs	((agenesis coronary) AND (hypoplastic coronary)) AND (clinical anatomy)	12	
CINHAL	((agenesis coronary) AND (hypoplastic coronary)) AND (clinical anatomy)	4	
WOS	((agenesis coronary) AND (hypoplastic coronary)) AND (clinical anatomy)	19	
Google Scholar	((agenesis coronary) AND (hypoplastic coronary)) AND (clinical anatomy)	22	
	Total	108	
